# The Changing Face of P300 BCIs: A Comparison of Stimulus Changes in a P300 BCI Involving Faces, Emotion, and Movement

**DOI:** 10.1371/journal.pone.0049688

**Published:** 2012-11-26

**Authors:** Jing Jin, Brendan Z. Allison, Tobias Kaufmann, Andrea Kübler, Yu Zhang, Xingyu Wang, Andrzej Cichocki

**Affiliations:** 1 Key Laboratory of Advanced Control and Optimization for Chemical Processes, Ministry of Education, East China University of Science and Technology, Shanghai, P.R. China; 2 Laboratory of Brain-Computer Interfaces, Institute for Knowledge Discovery, Graz University of Technology, Graz, Austria; 3 Department of Psychology I, University of Würzburg, Würzburg, Germany; 4 Laboratory for Advanced Brain Signal Processing, Brain Science Institute, RIKEN, Wako-shi, Japan; 5 Cognitive Neuroscience Laboratory, Department of Cognitive Science, University of California San Diego, La Jolla, California, United States of America; University of Houston, United States of America

## Abstract

**Background:**

One of the most common types of brain-computer interfaces (BCIs) is called a P300 BCI, since it relies on the P300 and other event-related potentials (ERPs). In the canonical P300 BCI approach, items on a monitor flash briefly to elicit the necessary ERPs. Very recent work has shown that this approach may yield lower performance than alternate paradigms in which the items do not flash but instead change in other ways, such as moving, changing colour or changing to characters overlaid with faces.

**Methodology/Principal Findings:**

The present study sought to extend this research direction by parametrically comparing different ways to change items in a P300 BCI. Healthy subjects used a P300 BCI across six different conditions. Three conditions were similar to our prior work, providing the first direct comparison of characters flashing, moving, and changing to faces. Three new conditions also explored facial motion and emotional expression. The six conditions were compared across objective measures such as classification accuracy and bit rate as well as subjective measures such as perceived difficulty. In line with recent studies, our results indicated that the character flash condition resulted in the lowest accuracy and bit rate. All four face conditions (mean accuracy >91%) yielded significantly better performance than the flash condition (mean accuracy = 75%).

**Conclusions/Significance:**

Objective results reaffirmed that the face paradigm is superior to the canonical flash approach that has dominated P300 BCIs for over 20 years. The subjective reports indicated that the conditions that yielded better performance were not considered especially burdensome. Therefore, although further work is needed to identify which face paradigm is best, it is clear that the canonical flash approach should be replaced with a face paradigm when aiming at increasing bit rate. However, the face paradigm has to be further explored with practical applications particularly with locked-in patients.

## Introduction

Brain-computer interface (BCI) systems allow for communication without movement. Users perform specific mental tasks that each has distinct patterns of brain activity. An artificial system categorizes these patterns to identify the associated mental tasks and thereby infer the messages or commands that the user meant to convey. Most modern BCIs rely on the EEG, which is recorded noninvasively via electrodes on the surface of the head [1]. While a variety of mental tasks have been explored for BCI control, most BCIs rely on motor imagery or visual attention ([2]–[3], [4], [5], [6], [7], [8]).

### “P300” BCIs

One type of BCI that usually relies on visual attention is called a P300 BCI ([9], for review, see [10], [11], [12]). This BCI is so named because it relies heavily on the P300, which is a well-known component of the event-related potential (ERP) that is largest when elicited by events that the subject considers important ([13], [14]). Therefore, subjects can generate larger, more distinct P300s by choosing to pay attention to specific events while ignoring others. For example, a user who wants to spell the letter “P” could silently count each time the “P” flashes while ignoring any flashes that do not include the “P”. If the BCI signal processing software correctly recognizes that the largest P300 is elicited whenever the “P” flashes, then the BCI system would spell the letter “P” on a monitor and move on to the next letter. Although spelling is the most common application, P300 BCIs have been used to control other applications such as a “BrainPainting” system, internet browser, robot arm, or environmental control ([15], [16], [17], [18]). Recently, different P300 BCI systems have even been implemented with standard assistive technology (AT) software for text entry, emailing, and internet surfing and evaluated by severely impaired end-users in terms of effectiveness (accuracy), efficiency (bit rate and subjective workload) satisfaction and other factors ([19], [20], [21]). The P300-BCI driven AT software proved effective and efficient was judged reliable and easy to learn, and users were satisfied with the BCI. However, the low information transfer rate limited the practical use of the system by end-users. Thus, improving speed while maintaining reliability is a major issue when bringing BCIs to the patients' bedsides [22].

P300 BCIs could be improved by enhancing the difference between attended and ignored events - which could entail more recognizable differences in the P300 and/or other components. In the past few years, different groups have focused on changes to stimulus presentation paradigms that generally seek to increase other components of the ERP that occur before or after the P300. P300 BCIs typically rely on not only the P300, but also other visual ERPs such as the N100, N200, and N400 components ([23], [24], [25], [26], [27], [28]).

For example, one study proposed a P300 BCI with stimuli that move instead of flash, which could elicit motion visual evoked potentials (M-VEPs) [29]. Hong et al. (2009) developed an offline M-VEP based spelling system, and showed that it might offer superior performance to a conventional P300 BCI [30]. Liu et al. (2010) validated the first online M-VEP BCI [31]. Jin et al. (2012) developed a combined system using moving flashes to improve the P300 BCI [32].

Another example of a study that focused on non-P300 components is Kaufmann et al. (2011b), which introduced stimuli that are transparently overlaid with famous faces. This approach accentuated the N170 and in particular the N400f, an ERP component involved in face recognition [28]. The resulting ERPs had a higher signal to noise ratio, which significantly improved classification accuracy. Zhang et al. (2012) reported that N170 and vertex positive potentials (VPP) also improve classification accuracy in a P300 BCI with stimuli that change to faces [33]. In addition to changing characters to faces, other types of changes may also offer advantages over the conventional “flash” approach in P300 BCIs ([25], [34], [35], [36], [37]).

The primary goal of this study was to parametrically compare a canonical “flash” condition with two other conditions in which the characters move or change to faces. Extending prior work, we explored both objective factors such as bit rate and classification accuracy as well as subjective factors such as whether subjects considered each condition difficult or tiring. This addressed the need for a direct, within-subject comparison of the two new types of stimulus changes that have gained the most attention in the literature (flashing, moving, and changing to faces), with consideration of personal preferences as well as performance. A secondary goal was to explore three new conditions in which the characters change to different types of faces. We hypothesized that faces that move and/or have an emotional expression could result in performance differences, with face stimuli leading to significantly better performance.

## Methods

### Subjects

Ten healthy subjects (6 male, aged 21–26 years, mean 24) participated in this study. All subjects signed a written consent form prior to this experiment and were paid for their participation. The local ethics committee approved the consent form and experimental procedure before any subjects participated. All subjects' native language was Mandarin Chinese, and all subjects were familiar with the Western characters used in the display. Four subjects had used a BCI before this study.

**Figure 1:**
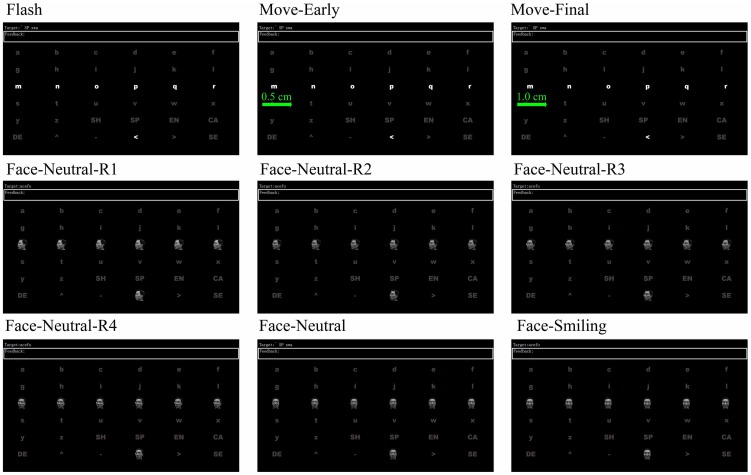
The display during the online runs. The five-letter target sequence is presented at the top of the screen, and the feedback is presented below it. Please see the text for a description of the different panels in this figure.

The face images were obtained by photographing the first subject. All subjects were familiar with this face, since subject 1 was a fellow student of subjects 2–9. This face was chosen partly because pilot testing using the same face as in Kaufmann et al., (2011) (Albert Einstein presenting his tongue) indicated that some subjects preferred the face of the first subject [28]. Subject 1 provided written consent to use his images within the study and in Figure 1 of this article, and understood that his face images would be published.

### Stimuli and procedure

After being prepared for EEG recording, subjects were seated about 105 cm in front of a monitor that was 30 cm high (visual angle: 16.3 degrees) and 48 cm wide (visual angle: 25.7 degrees). During data acquisition, subjects were asked to relax and avoid unnecessary movement. Figure 1 shows the display presented to all subjects, which was a 6×6 matrix with gray English letters and other characters against a black background. There were six conditions in the study, which differed only in the way that these characters changed. In different conditions, some of the characters would change by changing color, moving, and/or changing to different types of faces. We use the term “sub-trial” throughout this paper to refer to each individual event, such as a flash in the flash condition. The six conditions changed the stimuli as follows:

1. In the flash paradigm, the characters became white for 200 ms and then reverted to gray. (See Figure 1. Flash).
2. In the moving paradigm, each character became white and immediately moved 1 cm (visual angle: 0.5 degrees) to the right for 200 ms at a constant speed of 2.5 degrees/s. Next, the characters immediately reverted back to their original positions and reverted to gray. (See Figure 1. Flash, Move-Early and Move-Final).
3. In neutral face paradigm, each character changed to the “neutral face image” for 200 ms. (See Figure 1. Face-Neutral), and then reverted to gray characters.
4. In the smiling face paradigm, the only difference from the neutral face paradigm was that the “smiling face image” was used instead of the “neutral face image”. (See Figure 1. Face-Smiling).
5. In the shaking neutral face paradigm, the face rotated from the left to its front (see Figure 1. Face-Neutral-R1, Face-Neutral-R2, Face-Neutral-R3, Face-Neutral-R4 and Face-Neutral). Specifically, five images were shown, each for 25 ms, and each successive image was rotated 18 degrees toward the left side of the monitor. Hence, the rotation lasted 125 ms, after which the front face (see Figure 1. Face-Neutral) was shown for 75 ms.
6. In the shaking smiling face paradigm, the only difference from the shaking neutral face condition was the “smiling face image” was used instead of the “neutral face image”. (See Figure 1, Face-Smiling).

In all six conditions, after the 200 ms sub-trial, all characters reverted to their usual background state for 50 ms before the next flash began. Hence, in all conditions, there were four flashes per second.

Three of these conditions corresponded to conditions in our two previous papers ([28], [32]). The flash condition was similar to the flash condition in these papers, as well as the canonical flash condition in nearly all P300 BCI papers (e.g., [9], [23], [38]). The moving condition was similar to the “flash and move” condition in [32], and the neutral face condition was similar to the face conditions in [28]. The remaining three conditions modified this neutral face condition by modifying two independent variables: emotion (joy vs. neutral) and motion (motionless vs. shaking).

Instead of grouping the flashed characters into rows and columns, we developed an alternate flash pattern approach based on binomial coefficients ([35], [39]). In this paper, we used the set of *k* combinations (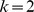) from set *n = 12*. Hence, there were twelve flash patterns, and between four and seven characters changed during each flash. Please see our prior work for additional details [25].

### Experiment set up, offline and online protocols

EEG signals were recorded with a g.USBamp and a g.EEGcap (Guger Technologies, Graz, Austria) with a sensitivity of 100 µV, band pass filtered between 0.1 Hz and 30 Hz, and sampled at 256 Hz. We recorded from EEG electrode positions Fz, Cz, Pz, Oz, F3, F4, P3, P4, P7, P8, O1, and O2 from the extended International 10–20 system.

In contrast to other P300 BCI approaches, the F3 and F4 channels were additionally selected since it was reported that F3 and F4 contain large N400 waves called fN400 [40].

The right mastoid electrode was used as the reference, and the front electrode (FPz) was used as a ground. Data were recorded and analyzed using the ECUST BCI platform software package developed through East China University of Science and Technology.

As noted, each **sub-trial** reflected each time a stimulus changed form a background stimulus, such as each flash in the flash condition. One **trial** contained all sub-trials with each of the twelve flash patterns. Since all conditions had 200 ms sub-trials followed by a 50 ms delay, each trial lasted three seconds. A **trial block** referred to a group of trials with the same target character. During offline testing, there were 16 trials per trial block. During online testing, the number of trials per trial block was variable, because the system adjusted this number to optimize performance as described in part 2.7. Each **run** consisted of five trial blocks, each of which involved a different target character. Subjects had a five minute break after each offline run, and a two minute break between each online run, since each online run was about more than four minutes shorter than each offline run.

There were six conditions, which were presented to each subject in pseudorandom order. For each condition, each subject first participated in three offline runs. Subjects had five minutes rest between each offline run. After the three offline runs, there were four online runs for each condition, which were presented in the same order as the offline runs. Each subject participated in the online and offline runs within each condition on the same day, and each subject participated in three conditions in each day to decrease fatigue. Hence, each subject participated in two **sessions** that each consisted of nine offline runs followed by twelve online runs.

At the beginning of each run, subjects viewed a 6×6 matrix containing gray English letters and other characters against a black background (see Figure 1). The top of the screen presented a five-character sequence containing the five targets for that run. The subject's task was to silently count each time the target character flashed. In the offline runs, subjects never received feedback. In the online runs, whenever the classifier identified the target character, the system displayed it in the “Feedback” line on the top left of the screen, then the run ended. After a run ended, the matrix remained on the screen without any changes for one second.

### Feature extraction procedure

A third order Butterworth band pass filter was used to filter the EEG between 0.1 Hz and 12 Hz. Although the P300 is primarily in the band 0.1–4 Hz [41], it can also be found in higher bands [42]. The EEG was downsampled from 256 Hz to 36.6 Hz by selecting every seventh sample from the filtered EEG. Single sub-trials lasting 800 ms were extracted from the data. The size of the feature vector was 12×29 (12 channels by 29 time points).

### Classification scheme

Bayesian linear discriminant analysis (BLDA) is an extension of Fisher's linear discriminant analysis (FLDA) that avoids over fitting. The details of the algorithm can be found in [43]. BLDA was selected because of its demonstrated classification performance in P300 BCI applications [43]. Data acquired offline were used to train the classifier using BLDA and obtain the classifier model. This model was then used in the online system.

### Practical bit rate

In this paper, we used two bit rate calculation methods called practical bit rate (PBR) and raw bit rate (RBR). The PBR is used to estimate the speed of the system in a real-world setting. Unless otherwise stated, all analyses in this paper are based on PBR; we only present RBR to facilitate comparisons with other studies. These two bit rate measures differ from each other in two ways. First, the PBR incorporates the fact that every error requires two additional selections to correct the error (a backspace followed by the correct character). The practical bit rate is calculated as RBR*(1–2*P), where RBR is the raw bit rate and P is the online error rate of the system [39]. Second, the RBR and PBR also incorporate the time between selections (1 second). Raw bit rate calculated with selection time was used to show the online information transfer rate of P300 BCIs which use other error correction methods [44] except “Backspace”.

### Adaptive system settings

The number of trials per average was automatically selected based on the classifier output. After each trial, the classifier would determine the target character based on data from all trials in that run block. If the classifier decided on the same character after two successive trials, then no new flashes were presented, and that character was presented as feedback to the subject.

For example, assume that the classifier decided that the letter “J” was the target based on the data from the first trial. The system would then present a second trial. The data from the first and second trials would be averaged, and the classifier would again decide which character was the target. If the classifier again selected “J”, then the BCI assumed that “J” was the correct target, and presented that feedback to the user. If the classifier did not select the “J”, then another trial would begin, and so on until the classifier chose the same letter two consecutive times or until 16 trial blocks elapsed. That is, after 16 trials with the same intended target letter, the classifier would automatically select the last output of classifier as the target character [45].

### Subjective report

After completing the last run, each subject was asked three questions about each of the six conditions. These questions could be answered on a 1–5 scale indicating strong disagreement, moderate disagreement, neutrality, moderate agreement, or strong agreement. The three questions were:

1. Was this paradigm annoying?
2. Did this paradigm make you tired?
3. Was this paradigm hard?

### Statistical analysis

Before statistically comparing classification accuracy and practical bit rate, data were statistically tested for normal distribution (One-Sample Kolmogorov Smirnov test) and sphericity (Mauchly's test). Consecutively, repeated measures ANOVAs with stimulus type as factor were conducted. Post-hoc comparison was performed with Tuckey-Kramer tests. The alpha level was adjusted according to Bonferoni-Holm with *α = .0083* (significant). Non-parametric Kruskal-Wallis tests were computed to statistically compare the questionnaire replies.

## Results

### Online performance

Table 1 shows the results from the online runs. Classification accuracy (F(5,45) = 7.78, p<.0001, η^2^ = 0.46) and practical bit rate (F(5,45) = 8.54, p<.0001, η^2^ = 0.49) were significantly different across the six conditions.

**Table 1:** Performance from online feedback runs.

		S1	S2	S3	S4	S5	S6	S7	S8	S9	S10	Average
Flash	Acc (%)	60	55	50	80	85	80	90	80	95	75	75±15.1
Move		65	90	75	95	90	95	80	85	90	75	84±9.9
N_face		90	80	85	100	100	100	90	100	85	95	92.5±7.5
S_Face		90	80	90	90	95	100	80	90	100	100	91.5±7.5
SN_Face		95	90	90	100	85	100	100	90	95	85	93±5.9
SS_Face		100	90	90	90	90	100	90	85	90	95	92±4.8
Flash	RBR (bit/min)	8.0	9.7	7.8	23.2	26.1	22.6	27.9	21.1	33.7	14.6	19.5±9.0
Move		9.8	29.9	15.4	26.4	25.0	26.1	22.8	23.3	26.2	14.5	21.9±6.5
N_face		25.4	24.0	28.1	46.0	29.1	38.3	34.9	25.2	27.6	30.8	30.9±6.9
S_Face		27.0	25.8	27.5	34.2	28.9	40.5	18.8	21.2	37.6	33.4	29.5±6.9
SN_Face		30.8	27.4	26.2	44.0	26.6	46.0	46.0	27.5	40.2	28.6	34.3±8.6
SS_Face		47.0	29.4	32.2	34.9	29.4	46.0	34.2	25.7	37.2	32.5	34.9±6.9
Flash	PBR (bit/min)	1.5	0.9	0	12.5	16.4	14.4	20.1	11.5	27.0	6.8	11.1±8.9
Move		2.7	21.4	7.1	21.7	18.2	21.4	12.3	14.8	19.0	6.7	14.5±7.0
N_face		18.4	12.9	17.5	40.0	26.6	34.1	24.5	23.1	17.2	25.0	23.9±8.2
S_Face		19.5	13.8	19.8	24.1	23.6	35.9	10.3	15.6	33.5	30.1	22.6±8.5
SN_Face		25.0	19.8	19.0	39	16.7	39.3	40.0	19.8	31.6	17.8	26.8±9.7
SS_Face		40.8	21.0	22.8	24.5	21.0	40.0	24.1	16.2	25.9	26.2	26.3±8.0
Flash	Avt	5.35	3.9	4.1	3.0	2.9	3.6	3.0	3.25	2.75	4.2	3.6±0.8
Move		5.0	2.8	4.0	3.5	3.35	3.6	3.05	3.25	3.2	4.25	3.6±0.7
N_face		3.3	2.8	2.7	2.3	3.6	2.7	2.4	4.1	2.75	3.0	3.0±0.6
S_Face		3.1	2.7	3.05	2.5	3.2	2.6	3.65	3.95	2.75	3.1	3.1±0.5
SN_Face		3.0	3.1	3.2	2.4	2.9	2.30	2.25	3.05	2.3	2.65	2.7±0.4
SS_Face		2.2	2.9	2.6	2.4	2.9	2.25	2.45	2.95	2.25	2.85	2.6±0.3

“Flash” is the flash-only paradigm, “Move” is moving-flash paradigm, “N_Face” is neutral face paradigm, “S_Face” is smiling face paradigm, “SN_Face” is neutral shaking face paradigm and “Face_SS” is smiling shaking face paradigm. “Acc” is classification accuracy, “RBR” is raw bit rate and “PBR” is practical bit rate (bits/min). “Avt” is average trials used for average.

A direct comparison of the three established paradigms within subjects (flash-only condition [9], moving-flash condition [32], and neutral face condition [27]), revealed significant differences for classification accuracy (F(2,18) = 9.61, p<.0015, η^2^ = 0.52) and practical bit rate (F(2,18) = 10.74, p<.0010, η^2^ = 0.54). Post-hoc comparison of the neutral face and flash-only condition showed significant differences in terms of classification accuracy (p<.0017) and significant differences in terms of PBR (p<.0083). No significant difference was found between the moving flash and flash-only condition as well as between moving flash and neutral face condition (all p>.0083; classification accuracy and PBR). However, average classification accuracy and both bit rate measures were larger for moving flash than flash-only (see Table 1).

In a separate comparison, we compared the four different face conditions to each other to explore the secondary goal of investigating the effect of movement and emotion in face stimuli. Neither comparison was significant (F(3,27) = 0.12, p = 0.9500, η^2^ = 0.01 for classification accuracy and F(3,27) = 0.80, p = 0.5000, η^2^ = 0.08 for practical bit rate).

### Non-P300 components

**Figure 2:**
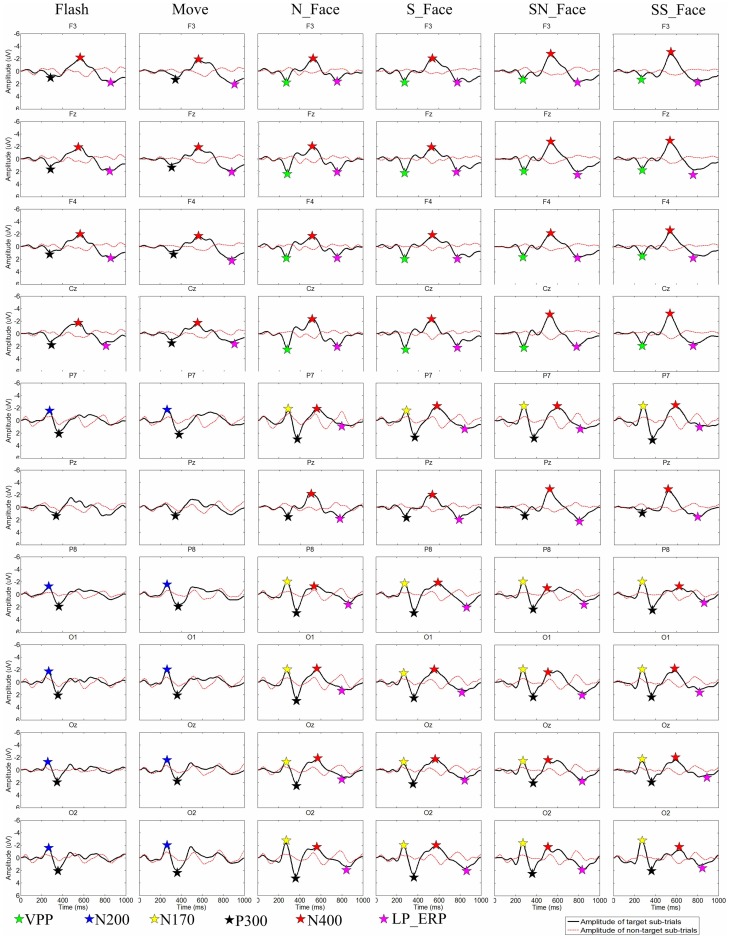
Grand averaged ERPs. Grand averaged ERPs across subjects 1–10 over sites F3, Fz, F4, Cz, P7, P8, O1, Oz and O2, LP_ERP is late positive ERP.

**Figure 3:**
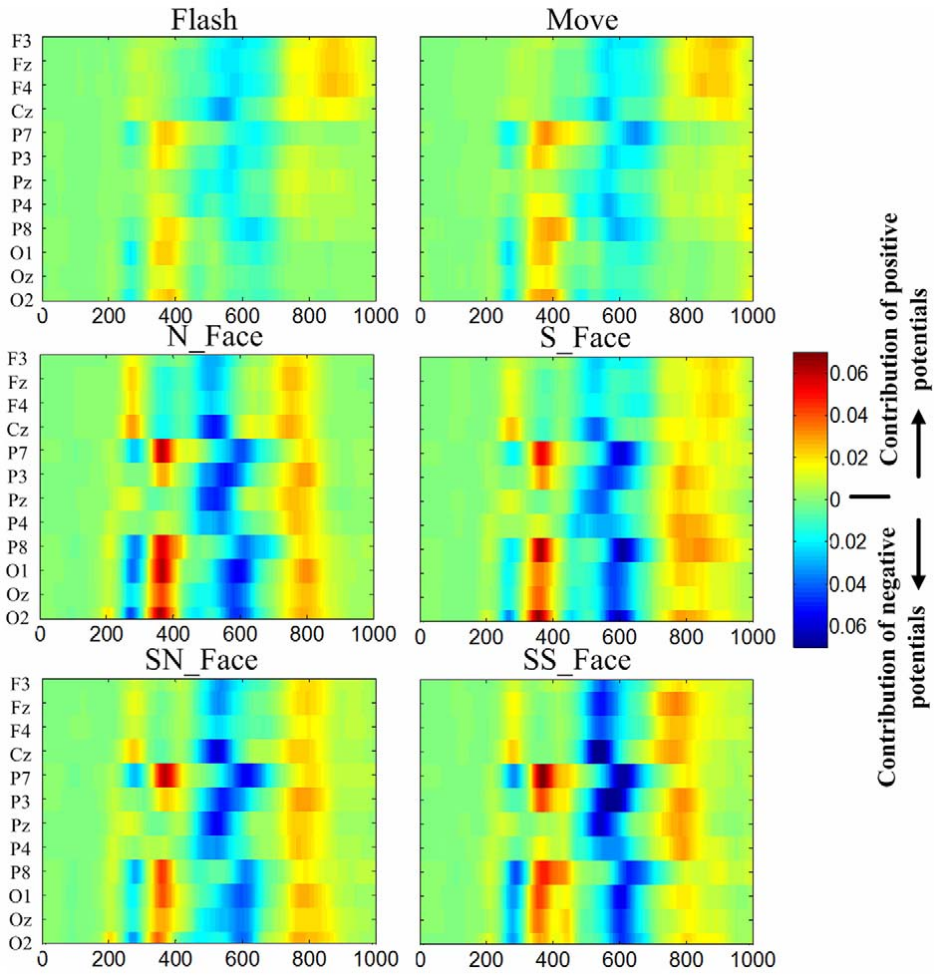
R-squared values of ERPs. R-squared values of ERPs from all paradigms at 0-1000ms averaged from subject 1–10 on site F3, Fz, F4, Cz, P7, P3, Pz, P4, P8, O1, Oz, O2.

**Figure 4:**
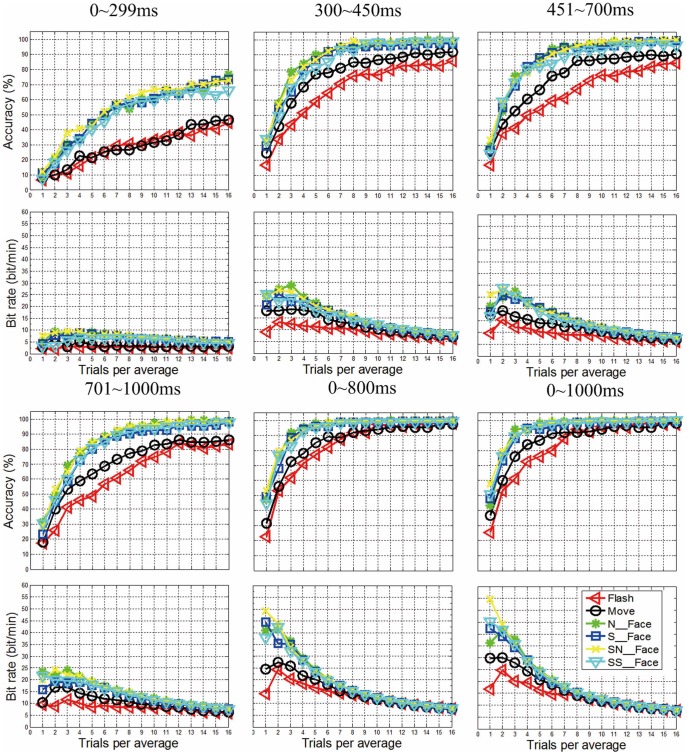
Classification accuracy and bit rate based on offline data. The two columns from left to right present the classification accuracy and bit rate based on offline data. The four rows from top to bottom present these measures based on data from 0–299 ms, 300–450 ms, 451–700 ms, 701–1000 ms, 0–800 ms and 0–1000 ms after flash onset, respectively.

Figure 2 shows that grand averaged ERPs across subjects 1–10 over sites F3, Fz, F4, Cz, P7, P8, O1, Oz and O2. Figure 3 and Figure 4 clearly indicate that non-P300 components play an important role in classification, as expected. Because this work entails numerous issues with different ERP components, we have submitted these results separately, as a companion paper. This paper includes a review of relevant literature, peak amplitude and latency measurements for different ERP components, statistical analyses, and discussion.

However, we did conduct another type of analysis within this study to explore the importance of different time periods within each ERP. We performed 15-fold cross validation with three offline data sets. Figure 4 shows the results obtained from data which was extracted from 0–299 ms, 300–450 ms, 451–700 ms, 700–1000 ms, 0–800 ms, and 0-1000 ms after flash onset. These results indicate that data before and after the P300 contribute to correct classification. This outcome supports our assertion that non-P300 components are underappreciated in so-called “P300” BCIs.

### Target to target interval

Target to target interval (TTI) is a critical determinant of P300 amplitude [46]. The TTI typically varies within P300 BCIs, and these variations can affect performance ([47], [48]). Furthermore, with very short TTIs, late components might actually reflect activity from subsequent trials.

In this study, very few ERPs were influenced by a second target stimulus that occurred during the 1000 ms after each target flash. With the flash pattern approach used here, the minimum TTI was 500 ms. Table 2 shows the distribution of TTIs. 99% of TTIs are longer than 750 ms, and 86% of TTIs are longer than 1000ms (see Table 2). This reflects that ERPs evoked by current stimulus are rarely influenced by the ERPs evoked by next stimulus.

**Table 2:** The percentages of TTIs between sub-trials.

TTI (ms)	500	750	1000	1250	1500	1750	2000	2250
(%)	0.42	12.9	13.1	16.9	12.9	17.1	13.3	13.3

### Subjective report

Table 3 presents the subjects' responses to the three questions. None of the participants reported any of the stimulus conditions as tiring, difficult or annoying, since they reported neutrality, moderate disagreement or strong disagreement with these questions. No significant difference was found between conditions (H(5) = 7.37, p = .19 for annoying; H(5) = 3.93, p = .56 for difficult; H(5) = 13.66, p = .02 for tired; adjusted alpha level: α = .0083).

**Table 3:** Subjects' responses to three questions for each of the six conditions.

		S1	S2	S3	S4	S5	S6	S7	S8	S9	S10	average
Flash	Tired	1	1	1	1	3	1	3	3	1	1	1.6±1.0
	Diff	1	2	1	1	1	1	1	1	1	1	1.1±0.3
	Annoy	1	1	1	1	1	1	2	1	1	1	1.1±0.3
Move	Tired	1	1	1	1	2	2	3	3	3	1	1.8±0.9
	Diff	1	1	1	1	2	2	2	1	1	1	1.3±0.5
	Annoy	1	1	1	1	1	1	3	1	1	1	1.2±0.6
N_Face	Tired	2	2	1	1	3	1	3	2	1	2	1.8±0.8
	Diff	1	2	1	1	1	1	2	1	1	1	1.2±0.4
	Annoy	1	1	1	2	1	1	2	1	1	1	1.2±0.4
S_Face	Tired	2	2	2	1	3	1	3	3	3	2	2.2±0.8
	Diff	2	1	1	1	1	1	2	1	1	2	1.3±0.5
	Annoy	2	1	1	1	1	1	2	1	1	2	1.3±0.5
SN_Face	Tired	2	2	3	1	2	1	1	3	2	2	1.9±0.7
	Diff	2	2	2	1	2	2	1	1	2	2	1.7±0.5
	Annoy	2	1	2	2	1	1	2	1	1	2	1.5±0.5
SS_Face	Tired	2	2	3	1	2	2	1	3	3	2	2.1±0.7
	Diff	2	2	2	1	2	1	2	1	2	2	1.7±0.5
	Annoy	2	1	2	2	1	1	2	1	1	2	1.5±0.5

Subjects were asked whether each condition seemed tiring, difficult, or annoying. Questions and responses are translated from Mandarin Chinese. A “1” means strong disagreement, a “2” means moderate disagreement, a “3” reflects a neutral response, a “4” means moderate agreement or a “5” means strong agreement.

## Discussion

The primary goal of this study was to compare two emerging stimulus approaches with each other and with a canonical “flash only” stimulus approach. In line with previous results, the moving-flash [32] and face stimuli [28] improved classification accuracy and bit rate relative to flash-only, and the face stimulus was the most effective approach. Our moving flash condition did not significantly differ from the flash only condition. However, we previously reported that the moving-flash condition was significantly better than the flash-only condition [32]. Unlike that study, we did not use a blue/green paradigm in the current study, and we did not improve the moving and flash times through pilot testing. Instead, to facilitate comparison to other conditions, we used a white/grey pattern, and the movement duration was as long as the flash duration. The present results suggest that these modifications might be crucial for further enhancing performance in the moving-flash condition.

The secondary goal was to compare an established face paradigm with three novel face paradigms. We hypothesized that manipulating facial emotion (smiling faces) or motion (shaking faces) might lead to more distinct ERPs and thereby improve performance. Although these manipulations did appear to influence the expected ERP components with some subjects, any ERP differences were not robust enough to lead to statistically significant performance differences within the face conditions.

However, the results do not necessarily discourage future research involving facial movement and/or emotion. For examples, angry faces elicit larger ERPs than happy faces [49], and inverted faces could evoke a larger N170 and thereby improve the performance of a BCI [33]. Other paradigmatic changes could improve performance by eliciting more ERP differences between attended and ignored stimuli.

For example, presenting new faces or other stimuli with each flash might enhance the P3a or other components ([14], [50], [51]). Presenting the same face each time could yield a small reduction in negative potentials, but could enhance positive potentials. The merits of novel face presentation should be explored in an online BCI study, since (at least) P300 habituation from offline non-BCI studies may differ from habituation in online studies with feedback ([24], [52]). Different instructions to subjects could also encourage more attention to faces and thus enhance face related components. For example, instead of counting stimuli, subjects could be asked to count only certain types of faces, such as happy, male, or familiar faces. These possibilities underscore that further research is needed to translate these speculative options into actual research results.

As none of the subjects considered any of the conditions especially tiring, annoying, or difficult, we conclude that the new stimulus material is feasible for BCI use. However, non-significant trends indicated a slight preference for simpler conditions, and that the smiling face condition was more tiring. These trends should be explored in further studies. Some types of subjects, such as elderly users or persons with attentional disorders, might have more trouble with some conditions.

This work is significant for two general reasons. First, we provide further support for an increasingly obvious conclusion: the canonical “flash-only” paradigm, which has dominated P300 BCIs for almost 25 years, does not outperform other approaches. Alternate paradigms are superior in terms of objectively measurable dependent variables like accuracy and bit rate, and do not differ significantly in terms of subjective report. Since these approaches can be easily implemented, there seems to be no reason to continue using “flash-only” P300 BCIs, specifically with patients. While this work helps elucidate the objective and subjective effects of different approaches, further research is needed to better identify which alternate approaches are most effective.

This work is also significant in drawing attention to the importance of non-P300 components. Consistent with earlier work, this article also shows that the “P300” BCI could be improved when other ERP components are included. Indeed, numerous distinct ERPs, both before and after the P300, can improve classification accuracy.

## Conclusion

This paper further investigated how P300 BCIs could be improved when combined with other ERPs. Face emotion and motion were not found to increase performance as compared to neutral face stimulation; however, faces significantly outperformed other stimulation approaches. Future work should further explore ways to enhance any differences between target and non-target flashes. This major challenge could entail not only ERP components but other differences such as alpha power ([23], [53]). Future work could also assess whether adding alternate tasks (such as imagined movement) can further improve performance in a hybrid BCI ([54], [55]). The next practical step should be implementing faces in ERP-based applications such as text entry, emailing and internet surfing with standard assistive technology [56].
